# Pretreatment TyG-BMI predicts CD19 CAR-T persistence and prognosis in diffuse large B-cell lymphoma

**DOI:** 10.3389/fonc.2026.1921922

**Published:** 2026-08-24

**Authors:** Zelin Liu, Ruijun Yang, Jiawen Chen, Ziyu Liu, Yangyang Ding, Keke Huang, Ya Liao, Linhui Hu, Lianfang Pu, Lujie Liu, Jiyu Wang, Qianshan Tao, Shudao Xiong

**Affiliations:** 1 Hematological Lab, The Second Affiliated Hospital of Anhui Medical University, Hefei, Anhui, China; 2 Department of Hematology, The Second Affiliated Hospital of Anhui Medical University, Hefei, Anhui, China; 3 Department of Hematology, Affiliated Hospital of Jiangnan University, Wuxi, Jiangsu, China; 4 Department of Hematology, The Second Affiliated Hospital of Nanchang University, Nanchang, Jiangxi, China; 5 Department of Hematology, The First Affiliated Hospital of Bengbu Medical College, Bengbu, Anhui, China; 6 Weifang Yidu Central Hospital, Qingzhou, Shandong, China

**Keywords:** CD19 CAR-T, diffuse large B cell lymphoma, Pre-treatment assessment, Prognostic model, Triglyceride glucose-body mass index

## Abstract

**Background:**

Although CD19 CAR-T cell therapy has improved outcomes in relapsed or refractory diffuse large B-cell lymphoma (DLBCL), responses are variable and predictive biomarkers are lacking. The triglyceride glucose-body mass index (TyG-BMI)—a surrogate of insulin resistance—has shown prognostic value in solid tumors, but its relevance to DLBCL and CAR-T efficacy remains unknown.

**Methods:**

We retrospectively analyzed 204 newly diagnosed DLBCL patients treated with rituximab-containing regimens, stratified by median TyG-BMI. Prognostic associations were assessed by Kaplan-Meier and multivariate Cox regression, and a nomogram was constructed. In an exploratory sub-analysis, 20 patients who received CD19 CAR-T therapy were assessed. CAR-T cell persistence was measured by flow cytometry one week post-infusion and correlated with pre-infusion TyG-BMI by Pearson analysis. CRS and ICANS were graded per ASTCT criteria. Post-CAR-T survival was compared by Kaplan-Meier analysis stratified by median pre-infusion TyG-BMI.

**Results:**

High TyG-BMI was independently associated with poorer PFS (HR, 2.146; 95% CI, 1.092–4.214; *P* = 0.027) and OS (HR, 2.531; 95% CI, 1.084–5.913; *P* = 0.032), and further stratified risk within IPI risk groups and across disease stages. A nomogram incorporating TyG-BMI, IPI, and bone marrow involvement predicted 1-, 3-, and 5-year OS (AUC: 0.886, 0.799, 0.772). In the CAR-T subcohort (*n* = 20), elevated pre-infusion TyG-BMI was significantly inversely correlated with CAR-T cell persistence at one week post-infusion (*r* = -0.570, *P* = 0.009). Patients with high pre-infusion TyG-BMI experienced inferior responses to CAR-T therapy, with shorter median PFS (5.0 *vs.* 19.5 months) and OS (39.0 *vs.* 81.0 months) compared with the low TyG-BMI group.

**Conclusion:**

TyG-BMI is an independent prognostic factor in DLBCL that refines IPI-based stratification, and elevated pre-infusion TyG-BMI is associated with attenuated CAR-T cell persistence and inferior outcomes. This readily available metabolic index may aid risk assessment and treatment planning in DLBCL.

## Introduction

Diffuse large B-cell lymphoma (DLBCL) is the most common subtype of non-Hodgkin lymphoma, characterized by its aggressive clinical behavior and heterogeneous prognosis (1, 2). Although rituximab-containing regimen has significantly improved the prognosis of DLBCL, approximately 10–15% of patients still experience primary refractory, and 20–30% face relapse (3). For patients with relapsed or refractory (R/R) disease, CD19-targeted chimeric antigen receptor (CAR) T-cell therapy has emerged as a transformative treatment modality (4); however, individual responses vary considerably, and the biological determinants of CAR-T efficacy—including the role of host metabolic status—remain poorly defined. The identification of accessible biomarkers that predict both frontline prognosis and CAR-T treatment outcomes therefore represents an unmet clinical need.

The International Prognostic Index (IPI) has long served as the standard tool for risk stratification in DLBCL, guiding treatment decisions based on clinical factors (5). However, the IPI is derived entirely from clinical parameters and does not incorporate biochemical or metabolic characteristics of the tumor or host (6–8). While much of the literature linking BMI alone to cancer prognosis pertains to hormone-dependent malignancies (breast, endometrial cancers) (9, 10), evidence specific to lymphoid neoplasms supports a distinct non-hormonal mechanism: in DLBCL, metabolic dysfunction--particularly insulin resistance rather than adiposity per se--drives chronic inflammation that modulates tumor biology through cytokine-mediated pathways (IL-6, TNF-alpha) and immune microenvironment alterations (11, 12). Composite indices capturing insulin resistance may therefore be more biologically relevant than BMI alone in non-hormone-dependent malignancies such as DLBCL (13). Consequently, it incompletely captures the biological heterogeneity of DLBCL, particularly with respect to the patient’s nutritional and metabolic status, which has been increasingly recognized as a determinant of treatment tolerance and long-term outcomes. These limitations have motivated efforts to develop refined prognostic tools that integrate clinical indices with readily measurable metabolic biomarkers.

Insulin resistance (IR) is a central feature of metabolic syndrome (MetS) and reflects a state of chronic systemic inflammation and metabolic dysregulation (14, 15). Mechanistically, IR promotes a pro-tumorigenic microenvironment in DLBCL through multiple interconnected pathways: hyperinsulinemia directly activates PI3K/Akt/mTOR signaling in malignant B cells, enhancing proliferation and survival (16); chronic hyperglycemia fuels aerobic glycolysis (the Warburg effect), providing metabolic substrates that sustain rapid tumor growth (17, 18); and IR-associated dyslipidemia alters cell membrane composition and lipid raft signaling, potentially modulating B-cell receptor activation and NF-kB pathway activity (19). The systemic inflammatory milieu of IR--elevated IL-6, TNF-alpha, and CRP--promotes immune evasion and T-cell exhaustion, directly relevant to CAR-T cell therapy efficacy (20). MetS has been independently associated with inferior survival in DLBCL (21), and IR—present in over half of DLBCL patients—correlates with elevated inflammatory markers including C-reactive protein (11, 22). Among available IR assessment methods, the triglyceride-glucose (TyG) index, derived from fasting plasma glucose and triglyceride levels, has been validated as a simple, indirect surrogate marker of IR (23, 24). TyG was selected over HOMA-IR for several reasons: first, HOMA-IR requires fasting insulin measurement not routinely performed in hematology/oncology practice and unavailable for most patients in our cohort (25); second, TyG demonstrates comparable or superior diagnostic accuracy to HOMA-IR while relying solely on routine biochemical parameters (fasting glucose and triglycerides) universally measured at diagnosis (26); third, unlike insulin levels which may be influenced by exogenous insulin therapy or beta-cell failure, TyG components remain stable across a wider range of clinical scenarios (27). Guerrero-Romero et al. demonstrated that TyG achieved an AUC of 0.858 for identifying insulin resistance when validated against the euglycemic-hyperinsulinemic clamp (gold standard), confirming its diagnostic accuracy as a surrogate IR marker (28). Notably, combining TyG with body mass index (BMI) yields the TyG-BMI index, which outperforms TyG alone in predicting adverse outcomes in cardiovascular disease (29, 30). While TyG-BMI has been primarily investigated in hypertension and coronary artery disease (31, 32), recent studies have demonstrated its prognostic relevance in pancreatic and gastrointestinal cancers (33, 34). However, the relationship between TyG-BMI and clinical outcomes in DLBCL—including its potential relevance to CD19 CAR-T cell therapy—has not been investigated.

In this study, we sought to determine whether TyG-BMI, measured at initial diagnosis, is associated with overall survival (OS) and progression-free survival (PFS) in newly diagnosed DLBCL patients treated with rituximab-containing regimens. We further aimed to develop a novel prognostic model integrating TyG-BMI with the IPI to improve risk stratification. In an exploratory sub-analysis, we evaluated whether pre-infusion TyG-BMI is associated with CAR-T cell persistence, adverse events, and post-CAR-T survival in a subset of patients who subsequently received CD19 CAR-T therapy for R/R disease.

## Methods

### Patients’ characteristics

A total of 204 new diagnosed DLBCL patients between February 2013 and June 2024 were enrolled from the Second Affiliated Hospital of Anhui Medical University. All clinical data were retrieved from the electronic medical records in the hospital database. Among them, 20 patients with relapsed or refractory disease subsequently received CD19-targeted CAR-T cell therapy. This study received approval from the institutional review board, and all participants provided written informed consent.

The inclusion criteria for patients were as follows: a) aged 16 years or older with a confirmed diagnosis of DLBCL according to the 2016 World Health Organization (WHO) criteria; b) who had fasting glucose and fasting triglycerides tested at diagnosis; c) without any malignancy other than lymphoma at the time of diagnosis; d) treated with a rituximab-containing treatment regimen (R-CHOP).

Exclusion criteria were defined as follows: a) patients with primary involvement of the central nervous system and transformed indolent lymphoma; b) individuals who had not received formal treatment; c) those with previous diagnosis and treatment at other hospitals, lacking complete information.

Bone marrow involvement and the extent of lymphoma cell infiltration are determined based on the results of the morphological examination and immunohistochemistry.

### Treatment

All patients received a minimum of six cycles of R-CHOP chemotherapy regimen (21 days per course). They were given rituximab intravenously at a dose of 375 mg/m^2^, cyclophosphamide at a dose of 750 mg/m^2^, epirubicin at a dose of 50 mg/m^2^, and vincristine at a dose of 3 mg/m^2^ on the first day of each cycle. Additionally, they received dexamethasone intravenously at a daily dosage of 15 mg for five consecutive days. Before treatment, conventional promethazine and dexamethasone were administered to prevent allergic reactions. We also collected the initial treatment regimen, observed the response of each patient to the initial treatment. Further details of the study workflow have been described in previous studies, including patient screening, CD19 CAR-T cell manufacturing, conditioning chemotherapy, CD19 CAR-T infusion, disease assessment, and follow-up (35).

### Clinical parameter indicators

We collected clinical information of DLBCL patients at the time of diagnosis, including age, sex, white blood cells, hemoglobin, platelets, total cholesterol, fasting blood glucose (FBG), triglycerides (TG), total cholesterol (TC), height, weight, B symptoms, Eastern Cooperative Oncology Group performance status (ECOG PS), lactate dehydrogenase (LDH) level, Ann Arbor stage, International prognostic score (IPI), BM tumor cell count. The date of death was obtained from the clinical records or by telephone calls to their relatives. We then calculated body mass index (BMI), TyG index, and TyG-BMI index. BMI was defined as weight (in kilograms)/height squared (in meters), TyG index was calculated using the formula Ln [TG (mg/dL) × FBG (mg/dL)/2], and TyG-BMI was computed by TyG index × BMI (28). For the purpose of group stratification, patients were dichotomized by the median TyG-BMI value (27.65) into high and low groups. When BMI is referenced independently, the WHO classification for Asian populations was applied: underweight (< 18.5 kg/m2), normal weight (18.5-23.9 kg/m2), overweight (24.0-27.9 kg/m2), and obese (≥ 28.0 kg/m2), consistent with Chinese obesity guidelines.

### Statistical analysis

All data were analyzed using SPSS (version 25.0) or R studio (version 3.4.1) software. Baseline characteristics were presented as percentages of the total for categorical variables or as mean value ± standard deviation of the mean (SD) for continuous variables. Categorical variables were tested for significance using Fisher’s exact test or χ^2^-test, as appropriate. Normally distributed values were compared between the low TyG-BMI groups and high TyG-BMI groups using unpaired Student’s t-tests. Non-normally distributed values were compared using Mann-Whitney tests between the low TyG-BMI groups and high TyG-BMI groups. The primary outcomes were OS and PFS. Survival curves were constructed using the Kaplan-Meier method, and any differences in survival were evaluated with a stratified log-rank test. The Hazard ratio (HR) and its corresponding 95% confidence interval (CI) were estimated using the Cox regression model with statistical significance set at *P* < 0.05. The prognostic nomogram was established by using the “rms” and “regplot” R packages, and the regression coefficients were used to evaluate the survival probability of 1-year, 3-year, and 5-year OS in DLBCL patients. The Harrell consistency index (C-index) was used to verify the performance of the nomogram. The area under the curve (AUC) compared risk stratification models’ predictive efficacy. For the correlation between TyG-BMI and CAR-T cell persistence in the exploratory subcohort (*n* = 20), both Pearson and Spearman correlation analyses were performed. The Shapiro-Wilk test confirmed that TyG-BMI values and CAR-T cell persistence data approximated a normal distribution in this subcohort (*P* > 0.05 for both variables); therefore, Pearson correlation was reported as the primary analysis, with Spearman correlation presented as a sensitivity analysis to ensure robustness. The correlation coefficient (*r*) was interpreted according to established benchmarks: |*r*| < 0.3 as weak, 0.3-0.7 as moderate, and > 0.7 as strong.

## Results

### Clinical and biochemical characteristics by TyG-BMI group

A total of 204 newly diagnosed DLBCL patients were included, with a median age of 61.73 years (range, 23–86 years). 26 patients (12.75%), 18 (8.82%), 38 (20.1%), and 122 (59.80%) were classified as Ann Arbor stage I, II, III, and IV, respectively. According to IPI criteria, 41 (20.10%), 40 (19.61%), 77 (37.75%), and 46 (22.55%) of patients were classified into low, low-intermediate, high-intermediate, and high-risk groups, respectively. After 2–4 cycles of chemotherapy, 139 patients (68.14%) achieved complete remission (CR). The median TyG-BMI level at diagnosis was 27.65. Patients were dichotomized into low and high TyG-BMI groups by the median. Significant differences between the two groups were observed in total cholesterol (*P* = 0.005), BMI (*P* < 0.001), TyG index (*P* < 0.001), Ann Arbor stage (*P* = 0.006), bone marrow involvement (*P* = 0.011), CR rate (*P* < 0.001), relapse rate (*P* < 0.001), and death (*P* < 0.001). Baseline clinical characteristics of the two groups are summarized in **Table 1**.

**Table 1:** Baseline clinical characteristics by TyG-BMI group.

Variables	Overall Mean ± SD (%) (*n* = 204, %)	Low TyG-BMI Mean ± SD (%) (*n* = 102, %)	High TyG-BMI Mean ± SD (%) (*n* = 102, %)	*P* Value
Age (years)	61.73 ± 12.12	61.16 ± 13.33	61.22 ± 11.94	0.730
Gender				0.203
Male	99 (48.53)	52 (50.98)	47 (46.08)	
Female	105 (51.47)	50 (49.02)	55 (53.92)	
WBC (×10^9^/L)	5.89 ± 3.49	6.31 ± 2.50	6.37 ± 4.38	0.342
Hb (g/L)	111.65 ± 24.43	112.93 ± 22.45	113.77 ± 25.86	0.480
PLT (×10^9^/L)	181.31 ± 108.74	200.36 ± 92.72	178.95 ± 134.69	0.199
TC (mmol/L)	4.07 ± 1.25	3.93 ± 1.14	4.39 ± 1.26	**0.005** ^a^
TyG (mmol/L)	1.26 ± 0.57	0.83 ± 0.36	1.68 ± 0.46	**< 0.001** ^a^
BMI (kg/m2)	22.81 ± 3.45	21.83 ± 3.07	23.90 ± 3.54	**< 0.001** ^a^
B symptoms				0.574
present	110 (53.92)	53 (51.96)	57 (55.88)	
absent	94 (46.08)	49 (48.04)	45 (44.12)	
ECOG PS				0.778
≥2	92 (45.10)	45 (44.12)	47 (46.08)	
<2	112 (54.90)	57 (55.88)	55 (53.92)	
LDH				0.161
abnormal	98 (48.04)	44 (43.14)	54 (52.94)	
normal	106 (51.96)	58 (56.86)	48 (47.06)	
Ann Arbor stage				0.006^a^
I	26 (12.75)	14 (13.73)	12 (11.76)	
II	18 (8.82)	10 (9.80)	8 (7.84)	
III	38 (18.63)	22 (21.57)	16 (15.69)	
IV	122 (59.80)	56 (54.90)	66 (64.71)	
IPI				0.115
Low	41 (20.10)	20 (19.61)	21 (20.59)	
Low-intermediate	40 (19.61)	26 (25.49)	14 (13.73)	
High-intermediate	46 (22.55)	20 (19.61)	26 (25.49)	
High	77 (37.75)	36 (35.29)	41 (40.20)	
BM involement				0.011^a^
present	53 (25.98)	18 (17.65)	35 (34.31)	
absent	151 (74.02)	80 (78.43)	67 (65.69)	
CR	139 (68.14)	84 (60.43)	55 (39.57)	**< 0.001** ^a^
Relapse	105 (51.47)	33 (31.43)	72 (68.57)	**< 0.001** ^a^
Death	70 (34.31)	18 (25.71)	52 (74.29)	**< 0.001** ^a^

SD, standard deviation; WBC, white blood cell; Hb, hemoglobin; PLT, platelet; TC, total cholesterol; TyG, triglyceride glucose index; BMI, body mass index; TyG-BMI, triglyceride glucose-body mass index; ECOG PS, Eastern Cooperative Oncology Group performance status; LDH, lactate dehydrogenase; IPI, International prognostic score; BM, bone marrow.

^a^ Statistically significant (*P* < 0.05). The bolded text indicates a statistically significant difference (P < 0.05).

### High TyG-BMI is associated with poor survival outcomes

**Figure 1:**
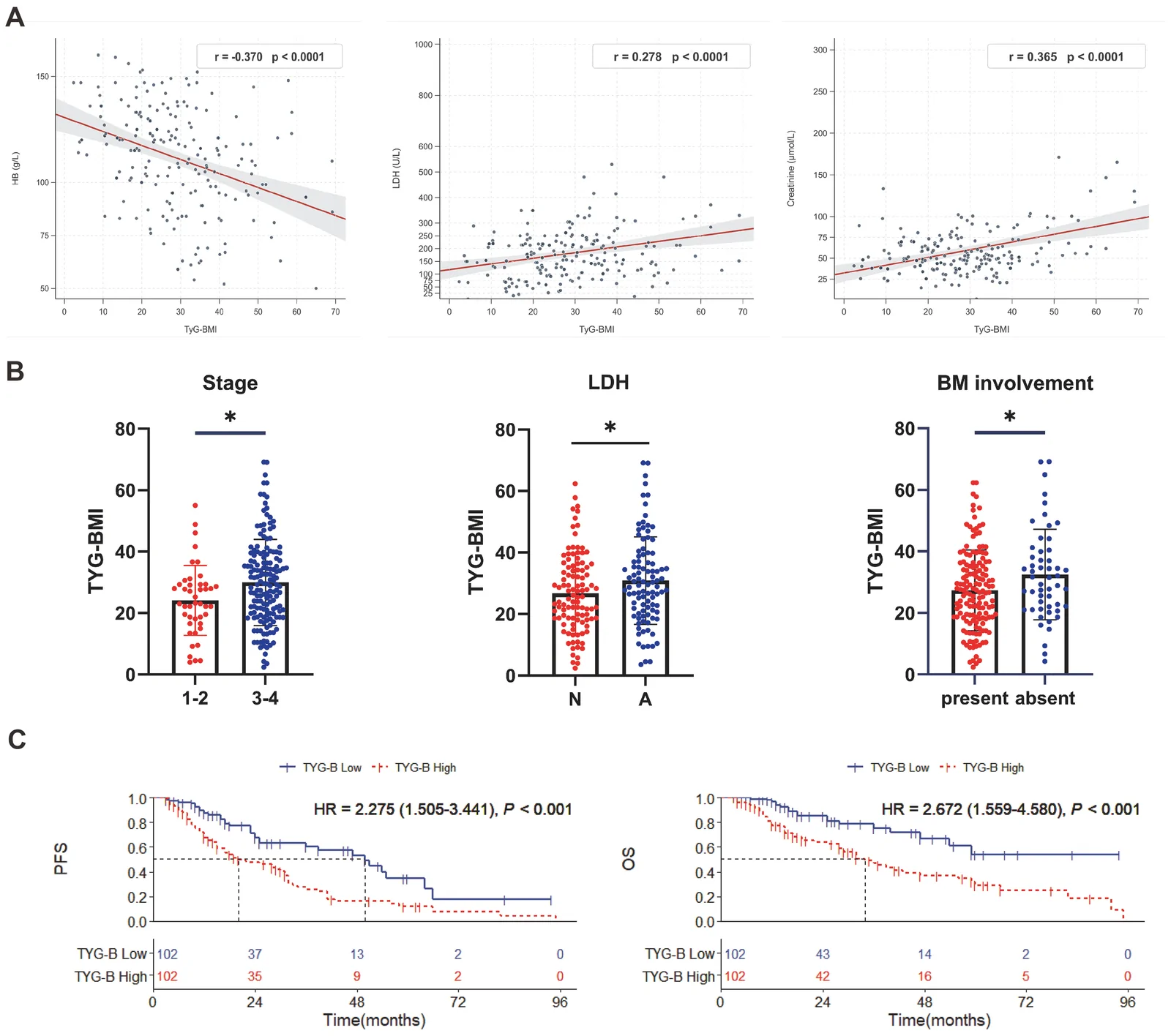
High TyG-BMI is associated with poor survival outcomes. **(A, B)** The correlation between the TyG-BMI index and clinical data. **(C)** Survival panels present the PFS curves (left) and OS curves (right). Statistical differences were calculated using the log rank test. *represents P < 0.05; **represents P < 0.01; *** for P < 0.001.

We first examined the correlation between TyG-BMI and clinical laboratory parameters. At initial diagnosis, TyG-BMI was negatively correlated with hemoglobin (*r* = -0.370, *P* < 0.001) and positively correlated with creatinine (*r* = 0.365, *P* < 0.001) and LDH (*r* = 0.278, *P* < 0.001; **Figure 1A**; **Table 2**). Subgroup analyses demonstrated that TyG-BMI levels were significantly associated with advanced disease stage, abnormal LDH, and bone marrow involvement (**Figure 1B**).

**Table 2:** TyG-BMI of newly diagnosed DLBCL patients was correlated with their laboratory data (*n* = 204) (*represents *P* < 0.05; **represents *P* < 0.01; *** for *P* < 0.001).

Clinical characteristics	*R*	*P* value
WBC	0.102	0.372
Neut%	0.212	0.421
Lymph%	-0.205	0.436
Mono%	-0.069	0.814
RBC	0.011	0.235
HB	-0.370	**< 0.001** ^a^
PLT	0.026	0.844
Albumin	-0.152	0.439
Globulin	0.294	0.473
A/G	-0.341	0.414
Creatinine	0.365	**< 0.001** ^a^
Uric acid	-0.042	0.452
Blood calcium	0.332	0.845
LDH	0.278	**< 0.001** ^a^
CRP	0.051	0.691
Cystatin	0.164	0.216

WBC, White blood cell; Neut %, Neutrophil Percentage; Lymph%, Lymphocyte Percentage; Mono%, Monocyte Percentage; RBC, Red Blood Cell; HB, Hemoglobin; PLT, Blood platelet; A/G, Albumin / Globulin; LDH, Lactate dehydrogenase; CRP, C-reactive protein.

^a^ Statistically significant (*P* < 0.05). The bolded text indicates a statistically significant difference (P < 0.05).

We subsequently performed survival and prognostic analyses. The median follow-up duration was 24.13 months (range, 3–95 months). Among the 204 patients, 105 (51.74%) experienced disease progression, and 70 (34.31%) died. Kaplan-Meier analysis showed that patients with high TyG-BMI had significantly inferior PFS (median, 20.0 *vs.* 50.0 months; log-rank *P* < 0.001) and OS (median, 34.0 months *vs.* not reached; log-rank *P* < 0.001) compared with those with low TyG-BMI (**Figure 1C**).

### TyG-BMI refines risk stratification within disease stage and IPI subgroups

**Figure 2:**
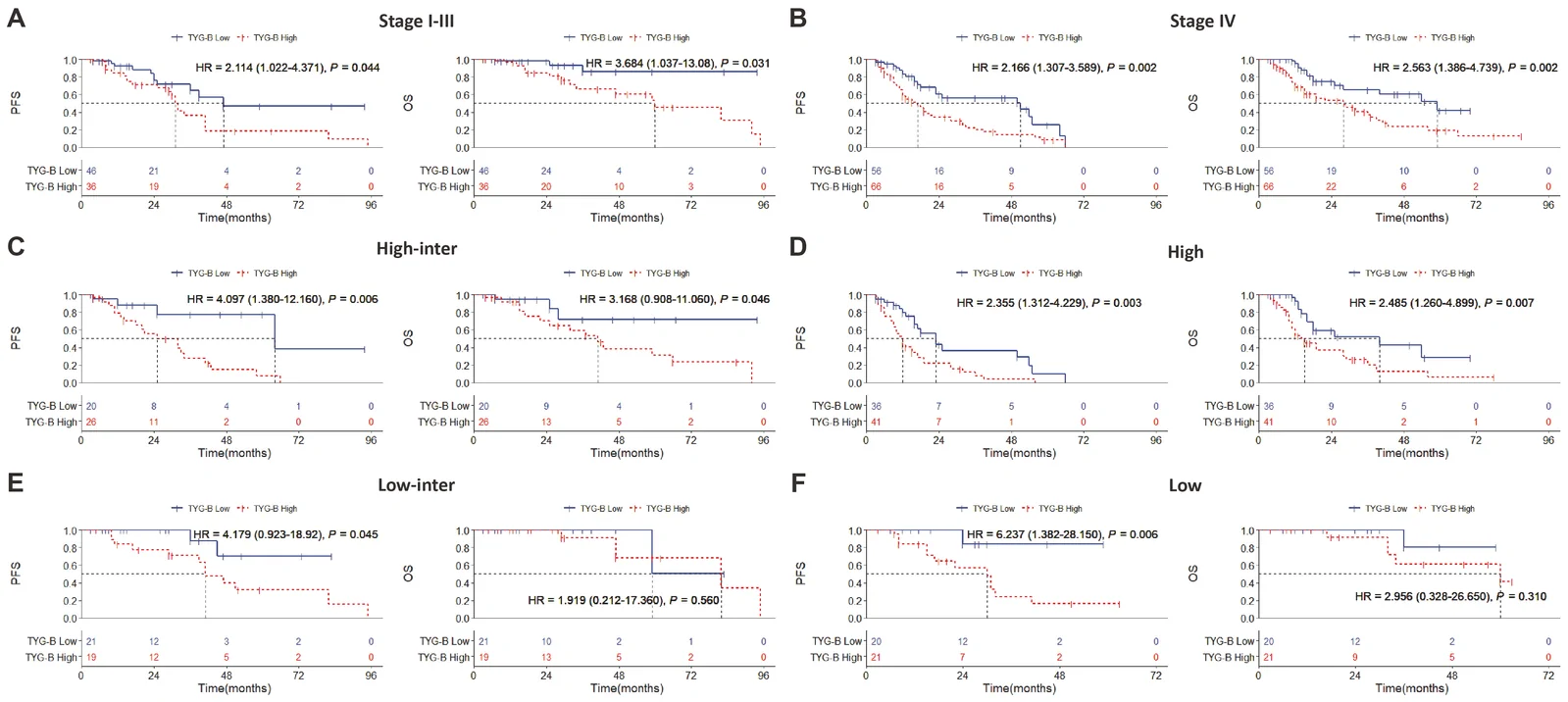
TyG-BMI refines risk stratification within disease stage and IPI subgroups. Kaplan-Meier survival curves according to the TyG-BMI of **(A)** patients of stage I-III; **(B)** patients of stage IV; **(C)** patients as the High-to-intermediate-risk group; **(D)** patients as the High-risk group; **(E)** patients as the Low-to-intermediate-risk group; **(F)** patients as the Low-risk group. Survival panels present the PFS curves (left) and OS curves (right). Statistical differences were calculated using the log rank test.

Given the association between TyG-BMI and clinical stage, we examined whether TyG-BMI could further stratify prognosis within different stage groups. Among patients with stage I-III disease, high TyG-BMI was significantly associated with inferior PFS (median, 31.0 *vs.* 47.0 months; log-rank *P* = 0.044) and OS (median, 60.0 months *vs.* not reached; log-rank *P* = 0.031; **Figure 2A**). A similar pattern was observed among stage IV patients for PFS (median, 17.0 *vs.* 50.0 months; log-rank *P* = 0.002) and OS (median, 28.0 *vs.* 59.0 months; log-rank *P* = 0.002; **Figure 2B**).

We further evaluated the prognostic value of TyG-BMI across IPI risk groups. In the high-intermediate and high-risk groups, patients with high TyG-BMI demonstrated significantly worse PFS (median, 25.0 *vs.* 64.0 months, log-rank *P* = 0.006; and 12.0 *vs.* 23.0 months, log-rank *P* = 0.003) and OS (median, 41.0 months *vs.* not reached, log-rank *P* = 0.046; and 15.0 *vs.* 40.0 months, log-rank *P* = 0.007; **Figures 2C, D**). In the low-intermediate and low-risk groups, high TyG-BMI was associated with inferior PFS (median, 41.0 months *vs.* not reached, log-rank *P* = 0.045; and 60.0 months *vs.* not reached, log-rank *P* = 0.006), but not OS (log-rank *P* = 0.560 and *P* = 0.310, respectively; **Figures 2E, F**). These findings indicate that TyG-BMI provides additional prognostic discrimination within the IPI framework. TyG-BMI retains prognostic value across age subgroups. To evaluate whether the prognostic effect of TyG-BMI differs by age, we performed age-stratified survival analyses. Among younger patients (age < 60 years, *n* = 79), high TyG-BMI was significantly associated with inferior PFS (log-rank *P* = 0.027; **Supplementary Figure S3A**) and OS (log-rank *P* = 0.035; **Supplementary Figure S3B**). Among older patients (age ≥ 60 years, *n* = 125), high TyG-BMI was similarly associated with worse PFS (log-rank *P* = 0.009; **Supplementary Figure S3C**) and OS (log-rank *P* = 0.018; **Supplementary Figure S3D**). Furthermore, in a multivariable Cox model incorporating a TyG-BMI x age interaction term, the interaction was not statistically significant for either PFS (*P* for interaction = 0.628; Concordance = 0.637; **Supplementary Figure S3E**) or OS (*P* for interaction = 0.474; Concordance = 0.652; **Supplementary Figure S3F**), indicating that the prognostic effect of TyG-BMI is consistent across age strata and not significantly modified by age.

### High TyG-BMI is associated with bone marrow involvement

**Figure 3:**
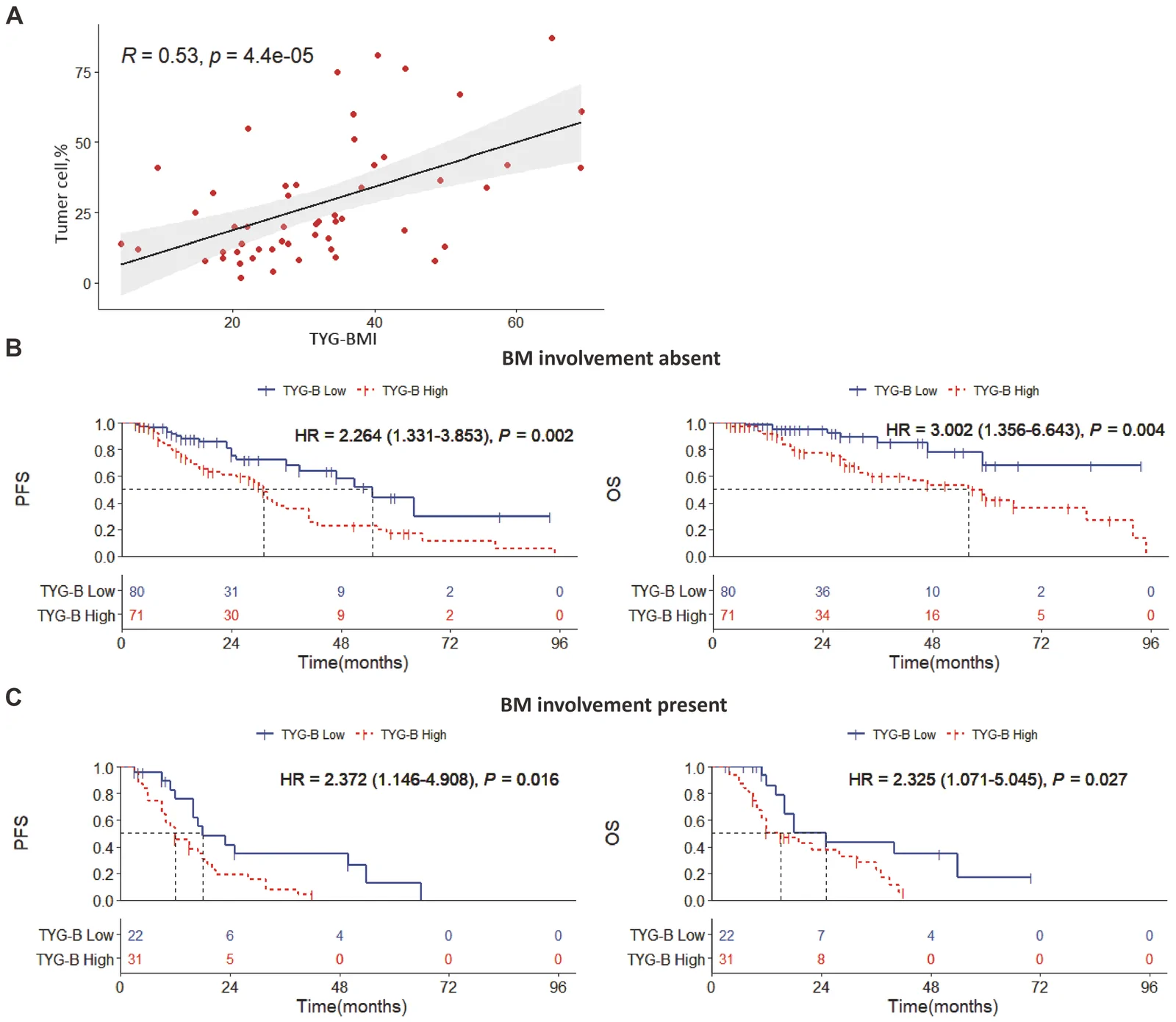
High TyG-BMI is associated with bone marrow involvement. **(A)** The TyG-BMI index is positively correlated with the percentage of tumor cells in the bone marrow. Kaplan-Meier survival curves according to the patients **(B)** with positive bone marrow involvement; **(C)** without bone marrow involvement. Survival panels present the PFS curves (left) and OS curves (right). Statistical differences were calculated using the log rank test.

We next assessed the relationship between TyG-BMI and bone marrow involvement. The TyG-BMI index was positively correlated with the number of bone marrow tumor cells (*r* = 0.53, *P* < 0.001; **Figure 3A**). Among patients with bone marrow involvement, those with high TyG-BMI had significantly worse PFS (median, 12.0 *vs.* 18.0 months; log-rank *P* = 0.016) and OS (median, 15.0 *vs.* 25.0 months; log-rank *P* = 0.027; **Figure 3B**). Even among patients without bone marrow involvement, high TyG-BMI remained associated with inferior outcomes (PFS: median, 56.0 months *vs.* not reached, log-rank *P* = 0.002; OS: median, 31.0 *vs.* 55.0 months, log-rank *P* = 0.004; **Figure 3C**), demonstrating that TyG-BMI retains prognostic value irrespective of bone marrow involvement status.

### TyG-BMI is an independent prognostic factor in newly diagnosed DLBCL

To identify factors independently associated with survival, we performed univariate followed by multivariate Cox regression analyses. In univariate analysis, variables significantly associated with PFS included age (≥60 years), PLT, TyG, TyG-BMI, ECOG PS (≥2), abnormal LDH, IPI (≥3), and bone marrow involvement; variables associated with OS included age (≥60 years), Hb, PLT, TyG, BMI, TyG-BMI, B symptoms, ECOG PS (≥2), abnormal LDH, stage (≥3), IPI (≥3), and bone marrow involvement. All variables reaching significance at P < 0.05 in univariate analysis were entered into the multivariate model. After adjusting for competing covariates, TyG-BMI (HR, 2.146; 95% CI, 1.092–4.214; P = 0.027) and bone marrow involvement (HR, 1.784; 95% CI, 1.114–2.858; P = 0.016) emerged as independent prognostic factors for PFS (**Table 3**). For OS, three variables retained independent significance: TyG-BMI (HR, 2.531; 95% CI, 1.084–5.913; P = 0.032), IPI (HR, 2.343; 95% CI, 1.166–4.707; P = 0.017), and bone marrow involvement (HR, 2.744; 95% CI, 1.450–5.190; P = 0.002; **Table 4**). Notably, TyG-BMI remained independently prognostic for both PFS and OS even when IPI—which itself incorporates age, stage, LDH, ECOG PS, and extranodal involvement—was included in the model, indicating that TyG-BMI captures prognostic information not accounted for by conventional clinical indices.

**Table 3:** Univariate and multivariate Cox-proportional hazard regression analyses predicting PFS in DLBCL patients.

Variables	Univariate analysis		Multivariate analysis	
	HR (95% CI)	*P* value	HR (95% CI)	*P* value
Age (≥60)	1.831 (1.201-2.793)	0.005^a^	1.474 (0.942-2.307)	0.090
Sex (male)	1.015 (0.687-1.498)	0.942		
WBC (continuous)	1.017 (0.963-1.074)	0.540		
Hb (continuous)	0.993 (0.985-1.001)	0.091		
PLT (continuous)	0.997 (0.995-0.999)	0.016^a^	0.999 (0.998-1.001)	0.589
TC (continuous)	1.180 (1.020-1.366)	0.262		
TyG (continuous)	2.105 (1.366-3.243)	0.001^a^	0.886 (0.439-1.787)	0.735
BMI ( continuous)	0.797 (0.531-1.197)	0.274		
TyG-BMI (continuous)	2.146 (1.415-3.254)	0.001^a^	2.146 (1.092-4.214)	**0.027** ^a^
B symptom (present)	1.464 (0.974-2.200)	0.067		
ECOG PS (≥2)	1.996 (1.349-2.954)	0.001^a^	1.242 (0.788-1.957)	0.352
LDH (abnormal)	2.483 (1.688-3.694)	< 0.001^a^	1.389 (0.773-2.497)	0.271
Stage (≥3)	1.736 (0.969-3.112)	0.064		
IPI (≥3)	2.756 (1.763-4.309)	< 0.001^a^	1.544 (0.964-2.473)	0.071
BM involvement	2.875 (1.921-4.304)	< 0.001^a^	1.784 (1.114-2.858)	**0.016** ^a^

WBC, white blood cell; Hb, hemoglobin; PLT, platelet; TC, total cholesterol; TyG, triglyceride glucose index; BMI, body mass index; TyG-BMI, triglyceride glucose-body mass index; ECOG PS, Eastern Cooperative Oncology Group performance status; LDH, lactate dehydrogenase; IPI, International prognostic score; BM, bone marrow. The bolded text indicates a statistically significant difference (P < 0.05).

^a^ Statistically significant (*P* < 0.05).

**Table 4:** Univariate and multivariate Cox-proportional hazard regression analyses predicting OS in DLBCL patients.

Variables	Univariate analysis		Multivariate analysis	
	HR (95% CI)	*P* value	HR (95% CI)	*P* value
Age ( ≥ 60)	1.706 (1.011-2.879)	0.045^a^	1.374 (0.783-2.412)	0.268
Sex (male)	1.157 (0.720-1.858)	0.546		
WBC (continuous)	1.009 (0.936-1.087)	0.816		
Hb (continuous)	0.991 (0.981-1.000)	0.049^a^	1.000 (0.998-1.003)	0.954
PLT (continuous)	0.997 (0.994-0.999)	0.013^a^	1.000 (0.998-1.003)	0.937
TC (continuous)	1.090 (0.910-1.306)	0.347		
TyG (continuous)	3.070 (1.730-5.450)	< 0.001^a^	1.157 (0.473-2.831)	0.749
BMI ( continuous)	0.502 (0.295-0.855)	0.011^a^	0.519 (0.282-0.954)	0.055
TyG-BMI (continuous)	2.778 (1.621-4.763)	< 0.001^a^	2.531 (1.084-5.913)	**0.032** ^a^
B symptom (present)	2.374 (1.397-4.036)	0.001^a^	0.776 (0.378-1.593)	0.490
ECOG PS ( ≥ 2)	2.307 (1.421-3.745)	0.001^a^	1.190 (0.676-2.094)	0.547
LDH (abnormal)	4.179 (2.462-7.094)	< 0.001^a^	1.500 (0.673-3.342)	0.322
Stage ( ≥ 3)	2.926 (1.265-6.767)	0.012^a^	1.068 (0.394-2.893)	0.897
IPI ( ≥ 3)	4.607 (2.414-8.795)	< 0.001^a^	2.343 (1.166-4.707)	**0.017** ^a^
BM involvement	4.410 (2.700-7.204)	< 0.001^a^	2.744 (1.450-5.190)	**0.002** ^a^

WBC, white blood cell; Hb, hemoglobin; PLT, platelet; TC, total cholesterol; TyG, triglyceride glucose index; BMI, body mass index; TyG-BMI, triglyceride glucose-body mass index; ECOG PS, Eastern Cooperative Oncology Group performance status; LDH, lactate dehydrogenase; IPI, International prognostic score; BM, bone marrow.

^a^ Statistically significant (*P* < 0.05). The bolded text indicates a statistically significant difference (P < 0.05).

### A nomogram integrating TyG-BMI improves survival prediction

**Figure 4:**
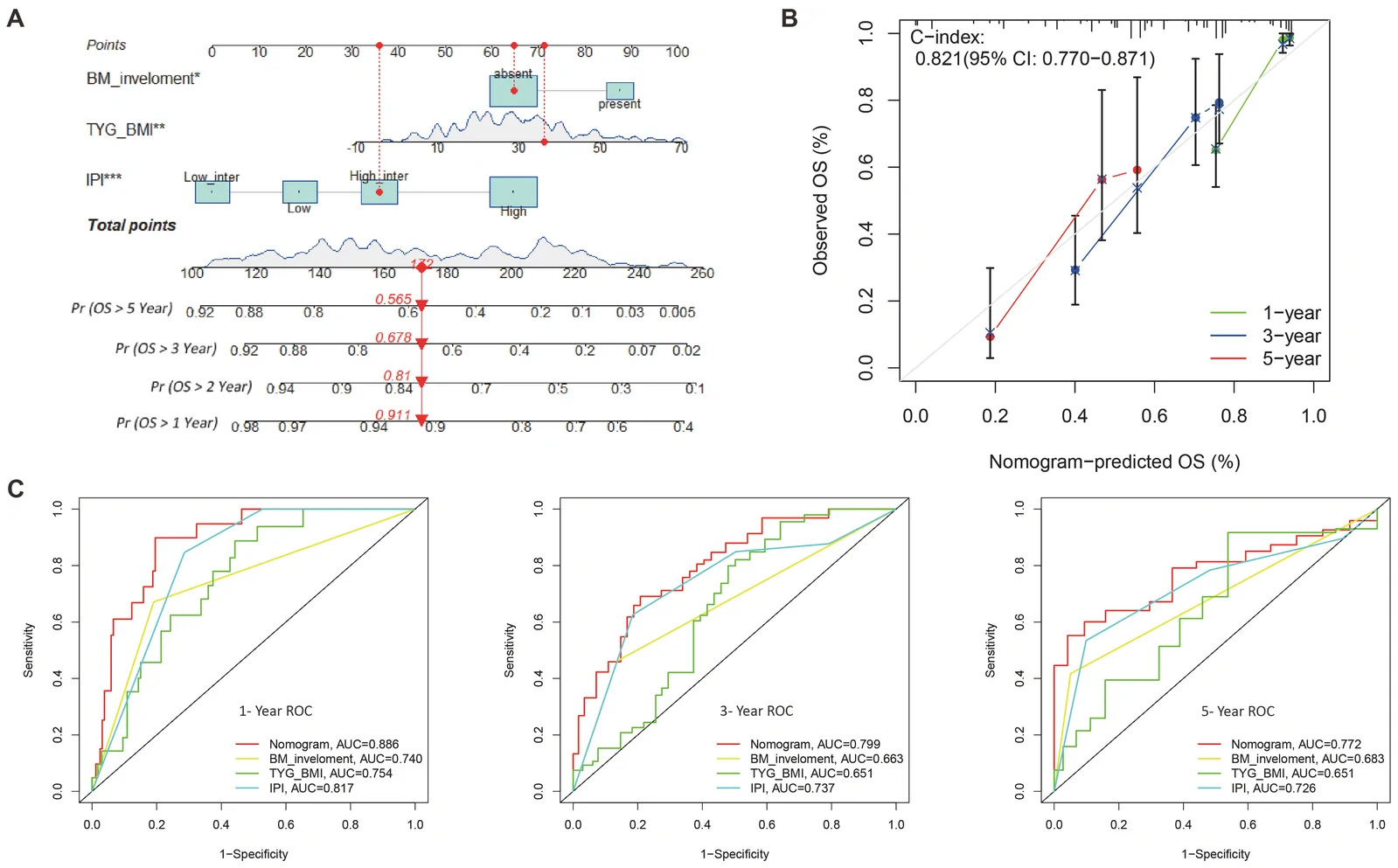
Evaluation of the new TyG index-IPI staging system for survival. **(A)** Nomogram based on patient age, TyG index, and IPI; The total score of the 3 variables is projected on the bottom scale to represent the probability of OS at 1-year, 3-year, and 5-year; **(B)** Calibration curve of 1-year, 3-year, and 5-year OS; **(C)** Comparison of ROC curves of different indicators for predicting 1-year, 3-year, and 5-year OS.

Given the independent prognostic value of TyG-BMI, we constructed a nomogram incorporating TyG-BMI, IPI, and bone marrow involvement to predict OS in DLBCL patients. Each patient was assigned a total score based on the regression coefficients of individual parameters, from which the predicted probabilities of 1-, 3-, and 5-year OS were derived (**Figure 4A**). The calibration curve demonstrated good agreement between predicted and observed survival, with a C-index of 0.821 (**Figure 4B**). The time-dependent AUC values for 1-, 3-, and 5-year OS were 0.886, 0.799, and 0.772, respectively, outperforming individual clinical indicators (**Figure 4C**). The nomogram also demonstrated favorable predictive performance for PFS, with AUC values of 0.757, 0.779, and 0.792 at 1, 3, and 5 years, respectively (**Supplementary Figure S1**).

### Elevated pre-infusion TyG-BMI is associated with attenuated CD19 CAR-T cell efficacy in DLBCL patients

Among the 204 patients, 20 (9.8%) with relapsed or refractory disease subsequently received CD19 CAR-T cell therapy and were included in a pre-specified exploratory sub-analysis. Baseline characteristics of the CAR-T subcohort, stratified by remission status, are presented in **Table 5**. The median age at CAR-T infusion was 51.5 years (range, 29–69 years), and 13 patients (65.0%) were male. After CAR-T infusion, 15 patients (75.0%) achieved remission, while 5 (25.0%) failed to respond.

**Table 5:** Baseline characteristics of DLBCL patients treated with CD19 CAR-T cells stratified by remission status.

Characteristic	Total (*n* = 20)	Remission (*n* = 15)	Non-remission (*n* = 5)
Age (years), mean ± SD	50.75 ± 12.53	49.47 ± 12.18	54.60 ± 14.19
Male, *n* (%)	13 (65.0%)	11 (73.3%)	2 (40.0%)
Female, *n* (%)	7 (35.0%)	4 (26.7%)	3 (60.0%)
Anthropometric parameters			
Height (cm), mean ± SD	167.79 ± 9.19	168.80 ± 9.76	164.00 ± 6.16
Weight (kg), mean ± SD	67.12 ± 14.42	70.95 ± 13.80	52.75 ± 3.30
BMI (kg/m²), mean ± SD	23.73 ± 3.93	24.79 ± 3.55	19.72 ± 2.55
Pre-infusion metabolic parameters			
TG (mmol/L), mean ± SD	1.48 ± 0.81	1.60 ± 0.77	1.13 ± 0.90
FBG (mmol/L), mean ± SD	5.63 ± 2.63	4.17 ± 2.55	6.01 ± 2.40
TyG index, mean ± SD	1.32 ± 0.60	0.45 ± 0.54	1.92 ± 0.62
TyG-BMI, mean ± SD	34.25 ± 16.28	26.54 ± 16.91	37.36 ± 13.36
Post-infusion parameters			
CAR-T cell survival rate (%), mean ± SD	58.07 ± 13.91	61.88 ± 14.21	47.40 ± 4.72
TG (mmol/L), mean ± SD	1.58 ± 0.69	1.51 ± 0.70	1.83 ± 0.67
FBG (mmol/L), mean ± SD	6.18 ± 2.01	5.96 ± 1.66	6.99 ± 3.21
TyG index, mean ± SD	1.45 ± 0.54	1.37 ± 0.54	1.74 ± 0.48
TyG-BMI, mean ± SD	35.03 ± 14.92	24.91 ± 16.78	35.36 ± 8.45
Clinical characteristics			
CRS grade, *n* (%)			
Grade 0	6 (30.0%)	5 (33.3%)	1 (20.0%)
Grade 1	7 (35.0%)	7 (46.7%)	0 (0.0%)
Grade 2	3 (15.0%)	1 (6.7%)	2 (40.0%)
Grade 3	4 (20.0%)	2 (13.3%)	2 (40.0%)
ICANS (any grade), *n* (%)	4 (20.0%)	1 (6.7%)	3 (60.0%)
Disease progression, *n* (%)	13 (65.0%)	8 (53.3%)	5 (100.0%)
PFS (months), mean ± SD	22.30 ± 20.03	25.87 ± 21.37	11.60 ± 10.78
OS (months), mean ± SD	40.25 ± 23.56	45.60 ± 24.41	24.20 ± 11.03
Death, *n* (%)	11 (55.0%)	6 (40.0%)	5 (100.0%)

Data are presented as mean ± SD for continuous variables and *n* (%) for categorical variables.

BMI, body mass index; TG, triglycerides; FBG, fasting blood glucose; TyG, triglyceride-glucose index; CRS, cytokine release syndrome; ICANS, immune effector cell-associated neurotoxicity syndrome; PFS, progression-free survival; OS, overall survival.

**Figure 5:**
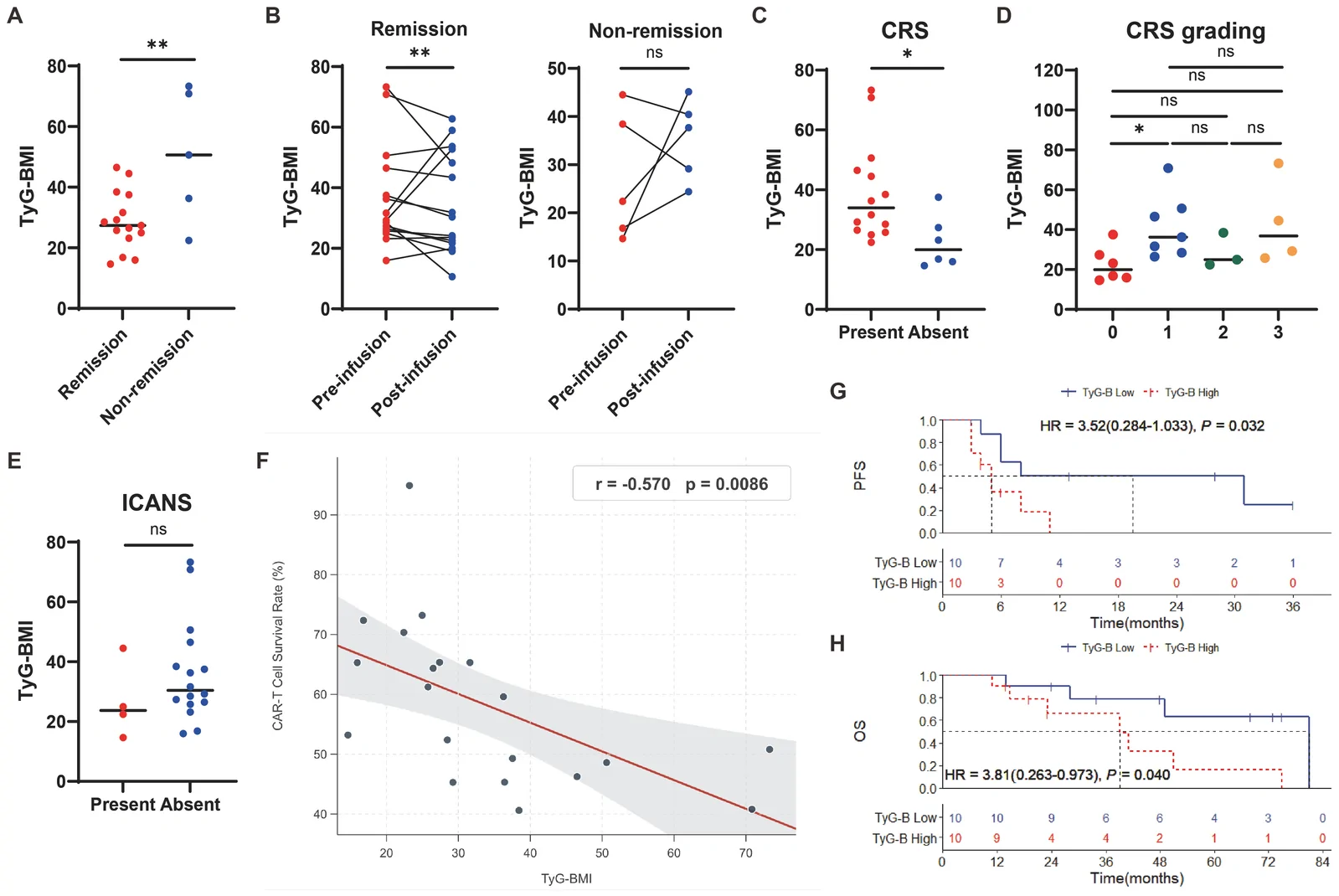
Elevated pre-infusion TyG-BMI is associated with attenuated CD19 CAR-T cell efficacy. **(A)** Pre-infusion TyG-BMI levels in patients who achieved remission versus those who did not. **(B)** Paired analysis of pre- and post-infusion TyG-BMI levels in the remission and non-remission groups. **(C)** Pre-infusion TyG-BMI levels stratified by CRS occurrence. **(D)** Pre-infusion TyG-BMI levels across CRS grades. **(E)** Pre-infusion TyG-BMI levels stratified by ICANS occurrence. **(F)** Pearson correlation between pre-infusion TyG-BMI and CAR-T cell survival rate at one week post-infusion. **(G, H)** Kaplan-Meier analysis of **(G)** PFS stratified by median pre-infusion TyG-BMI and **(H)** OS stratified by remission status in patients receiving CD19 CAR-T cell therapy. Statistical differences were calculated using the log rank test. *represents P < 0.05; **represents P < 0.01; *** for P < 0.001, ns for no statistical difference.

To examine whether pre-infusion metabolic status influences CAR-T treatment efficacy, we compared pre-infusion TyG-BMI levels between responders and non-responders. Patients in the non-remission group had significantly higher pre-infusion TyG-BMI levels than those in the remission group (**Figure 5A**). Paired analysis of pre- and post-infusion TyG-BMI levels within the remission group revealed a significant decrease following CAR-T infusion; this phenomenon was not observed in the non-remission group (**Figure 5B**).

We next investigated the relationship between pre-infusion TyG-BMI and CAR-T-related adverse events. Patients who developed CRS had higher pre-infusion TyG-BMI levels than those who did not (**Figure 5C**); however, no significant differences were observed across CRS grades (**Figure 5D**). Pre-infusion TyG-BMI levels did not differ significantly between patients with and without ICANS (**Figure 5E**).

To determine whether pre-infusion metabolic status affects CAR-T cell function, we correlated pre-infusion TyG-BMI with peripheral blood CAR-T cell persistence at one week post-infusion. Pearson analysis revealed a significant inverse correlation (*r* = -0.570, *P* = 0.009; **Figure 5F**). Pearson analysis revealed a significant inverse correlation (*r* = -0.570, *P* = 0.009; **Figure 5F**). A confirmatory Spearman rank correlation yielded consistent results (rho = -0.575, *P* = 0.011), supporting the robustness of this finding irrespective of distributional assumptions. The correlation coefficient (r = -0.57) indicates a moderate-strength association, accounting for approximately 32% of the variance in CAR-T cell persistence (R-squared = 0.325). While statistically significant, this moderate effect size indicates that other factors--such as tumor burden, T-cell intrinsic fitness, lymphodepletion intensity, and product characteristics--likely contribute substantially to CAR-T cell persistence variability. Interpretation should consider both the statistical significance and the moderate magnitude of the observed effect. Linear regression demonstrated that each one-unit increase in TyG-BMI was associated with a 0.48% reduction in CAR-T cell survival rate (*β* = -0.480, 95% CI, -0.821 to -0.139), suggesting that pre-existing metabolic dysfunction may compromise CAR-T cell expansion and persistence *in vivo*.

Finally, we performed survival analysis in the CAR-T subcohort, stratifying patients by median pre-infusion TyG-BMI. Patients in the high TyG-BMI group had inferior responses to CAR-T therapy, with more rapid disease relapse even among initial responders (median PFS, 5.0 *vs.* 19.5 months; *P* = 0.032; **Figure 5G**). Consistent with the differences in CAR-T cell persistence, clinical outcomes diverged markedly between the remission and non-remission groups (median OS, 81.0 *vs.* 39.0 months; *P* = 0.040; **Figure 5H**).

These exploratory findings provide preliminary evidence that pre-infusion TyG-BMI may be associated with CAR-T cell persistence and treatment outcomes in DLBCL; however, several important caveats temper this interpretation. The small sample size (*n* = 20, with only 15 responders and 5 non-responders) substantially limits statistical power, and the high dispersion observed in **Figure 5B**—particularly among non-remission patients—further underscores the imprecision of these estimates. Accordingly, these results should be regarded as hypothesis-generating rather than confirmatory, and the potential utility of TyG-BMI as a simple, readily available metabolic biomarker for predicting CAR-T outcomes requires rigorous validation in adequately powered prospective cohorts before any clinical application can be considered.

## Discussion

In this study, we demonstrate that elevated TyG-BMI is an independent poor prognostic factor in newly diagnosed DLBCL, with the capacity to further stratify risk within IPI subgroups and across disease stages. We constructed a nomogram integrating TyG-BMI, IPI, and bone marrow involvement that outperformed individual clinical indicators in predicting long-term survival. Notably, in a pre-specified exploratory analysis of patients who subsequently received CD19 CAR-T cell therapy, we found that higher pre-infusion TyG-BMI was significantly associated with attenuated CAR-T cell persistence and inferior post-CAR-T outcomes. These findings identify TyG-BMI as a simple, cost-effective metabolic biomarker with potential utility across the DLBCL treatment continuum—from frontline risk stratification to CAR-T therapy planning.

Metabolic syndrome (MetS), driven principally by insulin resistance (IR), has been increasingly implicated in the pathogenesis and prognosis of hematological malignancies (36–38). IR promotes a state of chronic, low-grade inflammation characterized by elevated circulating levels of pro-inflammatory cytokines and growth factors, including insulin-like growth factor-1 (IGF-1), which can stimulate the proliferation of malignant lymphocytes through receptor-mediated signaling (39–41). In DLBCL, IR is present in over half of cases and independently predicts inferior OS (11). Conceptually, MetS functions both as a modifier of treatment response—influencing chemotherapy tolerance and immunotherapy efficacy—and as an independent prognostic factor reflecting underlying tumor biology (21, 42). These roles are not contradictory but complementary: IR independently predicts inferior outcomes partly because it compromises treatment efficacy, and partly because it reflects more aggressive disease biology characterized by enhanced tumor proliferation and immune evasion (12). The term “independently associated” in this context indicates that after adjusting for established clinical variables (IPI, stage, LDH), MetS retains additional prognostic information through its dual impact on host treatment capacity and tumor microenvironment (43). These observations highlight the clinical relevance of metabolic dysfunction in lymphoma biology and underscore the need for accessible biomarkers that capture this dimension of host-tumor interaction (44).

The TyG-BMI index, which integrates fasting glucose and triglyceride levels with body mass, has emerged as a robust surrogate for IR that outperforms the TyG index alone (45, 46). In this study, while no statistically significant difference in survival outcomes was observed between patients with high and low TyG (median survival time PFS: 38.0 *vs.* 28.0 months, log-rank *P* = 0.057; median survival time OS: 59.0 *vs.* 41.0 months, log-rank *P* = 0.038; **Supplementary Figure 2**), but TyG-BMI exhibited strong prognostic predictive power, consistent with Wang et al.’s findings in pancreatic cancer research (33). Yang et al. further confirmed that TyG-BMI surpasses TyG in predicting and diagnosing esophageal cancer (47). We found that TyG-BMI exhibited stronger prognostic power than TyG alone in DLBCL, likely because the incorporation of BMI accounts for the contribution of obesity to IR-driven chronic inflammation and lipid dysregulation (48, 49). In our cohort, TyG-BMI was negatively correlated with hemoglobin and positively correlated with LDH and creatinine—three laboratory parameters with established prognostic significance in aggressive lymphomas—further supporting the link between metabolic dysfunction and adverse disease biology.

Bone marrow involvement is a well-established adverse prognostic factor in DLBCL (50). Our finding that TyG-BMI correlated positively with the percentage of bone marrow tumor cells (*r* = 0.53, *P* < 0.001) suggests a potentially bidirectional relationship: IR-driven systemic inflammation may facilitate bone marrow niche remodeling that supports lymphoma cell survival, while tumor infiltration of the bone marrow may itself exacerbate metabolic dysregulation through paracrine effects on adipocytes and stromal cells (51–53). The robust prognostic discrimination achieved by TyG-BMI in patients both with and without bone marrow involvement further supports its utility as a broad-spectrum risk indicator.

A central contribution of our study is the demonstration that TyG-BMI refines risk stratification within the IPI framework. The IPI, while widely used, relies solely on clinical parameters and does not account for the biological heterogeneity conferred by host metabolic status (6, 54). In our analysis, high TyG-BMI identified patients with significantly inferior PFS even within low and low-intermediate IPI groups, and further discriminated survival outcomes in high-intermediate and high-risk groups. These findings support the integration of metabolic biomarkers into existing prognostic models. Accordingly, the nomogram we developed—combining TyG-BMI, IPI, and bone marrow involvement—demonstrated superior predictive accuracy (C-index, 0.821; 5-year OS AUC, 0.772) compared with individual indicators, providing a clinically practical tool for personalized risk assessment.

A particularly important and novel finding of this study pertains to the CD19 CAR-T subcohort. CD19-directed CAR-T cell therapy has transformed the treatment landscape for R/R DLBCL; however, durable responses are achieved in only a subset of patients, and clinically accessible biomarkers predictive of CAR-T outcomes remain largely lacking. To our knowledge, this is the first study to evaluate the relationship between pre-infusion metabolic status—as reflected by TyG-BMI—and CAR-T cell function. We observed a significant inverse correlation between pre-infusion TyG-BMI and CAR-T cell persistence in peripheral blood at one week post-infusion (*r* = -0.570, *P* = 0.009), as well as a markedly shorter median PFS in patients with high TyG-BMI (5.0 *vs.* 19.5 months). Several mechanisms may underlie this association. First, IR-associated chronic inflammation, characterized by elevated IL-6, TNF-α, and other pro-inflammatory cytokines, is known to drive CAR-T-related CRS and may simultaneously promote T-cell exhaustion through chronic antigen-independent activation (55, 56). Consistent with this, we observed that patients who developed CRS had higher pre-infusion TyG-BMI levels. Second, the systemic metabolic milieu associated with IR—including hyperglycemia, hyperlipidemia, and oxidative stress—may directly impair CAR-T cell expansion, effector function, and memory formation. Third, the observation that TyG-BMI decreased significantly following CAR-T infusion in responders, but not in non-responders, raises the intriguing possibility that effective CAR-T therapy may partially reverse metabolic dysfunction, perhaps through the elimination of tumor-derived factors that drive systemic IR. Collectively, these findings suggest that pre-infusion TyG-BMI may serve as a readily available biomarker for identifying patients at risk of poor CAR-T outcomes and, potentially, for guiding metabolic optimization strategies prior to CAR-T therapy. It should also be noted that the median age of the CAR-T subcohort (51.5 years) was considerably younger than that of the overall cohort (61.73 years), likely reflecting clinical selection bias as younger patients with better performance status are preferentially referred for CAR-T therapy. This age difference may influence generalizability, given that immune reconstitution capacity, T-cell fitness, and baseline metabolic status differ between younger and older patients. Whether the observed TyG-BMI-CAR-T association applies equally to older DLBCL patients remains to be established. Regarding the interpretation of the correlation between TyG-BMI and CAR-T persistence, the observed Pearson *r* of -0.570 (confirmed by Spearman rho = -0.548) represents a moderate-strength association according to Cohen’s benchmarks. This magnitude indicates that TyG-BMI explains approximately one-third of the variance in early CAR-T persistence (*R*-squared = 0.325), leaving the majority of variability attributable to other biological determinants such as tumor burden, product composition, lymphodepletion adequacy, and T-cell intrinsic fitness. Accordingly, TyG-BMI should be viewed as one component of a multifactorial prognostic assessment rather than a standalone predictor.

In terms of clinical translation, the TyG-BMI index offers several practical advantages: it is derived from routinely measured laboratory parameters (fasting glucose, triglycerides) and anthropometric data (BMI), requires no specialized equipment or additional cost, and can be calculated at any point during the disease course. For frontline therapy, TyG-BMI may identify patients who warrant closer surveillance or more intensive treatment despite favorable IPI scores. For CAR-T therapy, pre-infusion TyG-BMI assessment could inform patient selection, toxicity risk stratification, and perhaps the timing of metabolic intervention. An important consideration is the potential confounding effect of intravenous dexamethasone (15 mg daily for five consecutive days) administered as part of R-CHOP. Glucocorticoids induce transient insulin resistance, hyperglycemia, and lipid metabolism alterations (57). However, TyG-BMI was calculated from fasting values obtained at diagnosis prior to chemotherapy initiation, and all patients received identical dexamethasone dosing, meaning glucocorticoid-induced metabolic effects were distributed equally across both TyG-BMI groups without systematically biasing the comparison. Nevertheless, longitudinal TyG-BMI fluctuations during treatment--potentially influenced by repeated glucocorticoid exposure--were not captured and represent an area for future investigation.

Our study has several limitations. First, this is a single-center retrospective analysis, which may introduce selection bias and limits generalizability. Second, the CAR-T subcohort is small (*n* = 20), With only 5 non-responders; The observed correlation, while statistically significant, should be regarded as preliminary evidence warranting confirmation rather than as a definitive finding establishing predictive utility; validation in larger, prospective CAR-T cohorts is essential. Third, whether interventions aimed at lowering TyG-BMI—such as lifestyle modification, metformin, or other insulin-sensitizing agents—can improve prognosis or CAR-T outcomes in DLBCL remains to be investigated in interventional studies. Fourth, the prognostic value of TyG-BMI in the context of other emerging DLBCL therapies, including bispecific antibodies and antibody-drug conjugates, was not assessed in this study and warrants future exploration. Fifth, although TyG-BMI was measured prior to treatment initiation, repeated dexamethasone exposure during R-CHOP may have transiently altered metabolic parameters during the treatment course; the impact of treatment-induced metabolic changes on outcomes warrants further study. Sixth, post-induction management (consolidation radiotherapy or additional chemotherapy) was not systematically recorded and could not be formally adjusted for. Although frontline R-CHOP was standardized and consolidation decisions were largely guided by variables already included in the multivariate model, we cannot exclude residual confounding from differential post-induction therapy.

## Conclusion

In this exploratory study, TyG-BMI emerged as a potentially useful predictor of outcomes in newly diagnosed DLBCL, and its integration may help refine IPI-based risk stratification. Our preliminary findings also suggest that elevated pre-infusion TyG-BMI is tentatively associated with attenuated CD19 CAR-T cell persistence and inferior post-CAR-T outcomes. These observations raise the possibility that TyG-BMI, as a simple and readily available metabolic parameter, could complement existing tools for frontline prognostication and pre-CAR-T risk assessment in DLBCL. However, given the exploratory nature of this analysis, these results should be interpreted with caution and require validation in larger, prospective cohorts before clinical application can be considered.

## Data Availability

The raw data supporting the conclusions of this article will be made available by the authors, without undue reservation.
