# Supplementary material for: Pretreatment TyG-BMI predicts CD19 CAR-T persistence and prognosis in diffuse large B-cell lymphoma
Source: Front Oncol. 2026 Aug 24;16:1921922. doi: 10.3389/fonc.2026.1921922 (PMC13547084; doi:10.3389/fonc.2026.1921922)
Supplement: Supplementary file 1 [file DataSheet1.docx]

**Supplyment Fig. 1.** Comparison of ROC curves of different indicators for predicting 1-year, 3-year, and 5-year PFS.

**Supplyment Fig. 2.** Kaplan-Meier survival curves according to the TyG of all patients. Survival panels present the PFS curves (left) and OS curves (right). Statistical differences were calculated using the log rank test.

**Supplyment Fig. 3.** (**A-D**) Kaplan-Meier survival curves according to the TyG of different age groups. (**E, F**) Multivariable Cox model incorporating a TyG-BMI x age interaction term. Survival panels present the PFS curves (left) and OS curves (right). Statistical differences were calculated using the log rank test.
